# Supplementary material for: Acute Shear Stress Induces TWIST-Mediated EndMT in Venous Endothelial Cells and Human Long Saphenous Veins
Source: Cells. 2025 Sep 2;14(17):1369. doi: 10.3390/cells14171369 (PMC12428368; doi:10.3390/cells14171369)
Supplement: Supplementary file 1 [file cells-14-01369-s001.zip › cells-3713546-supplementary.pdf]

Supp. Figure S1

(A)

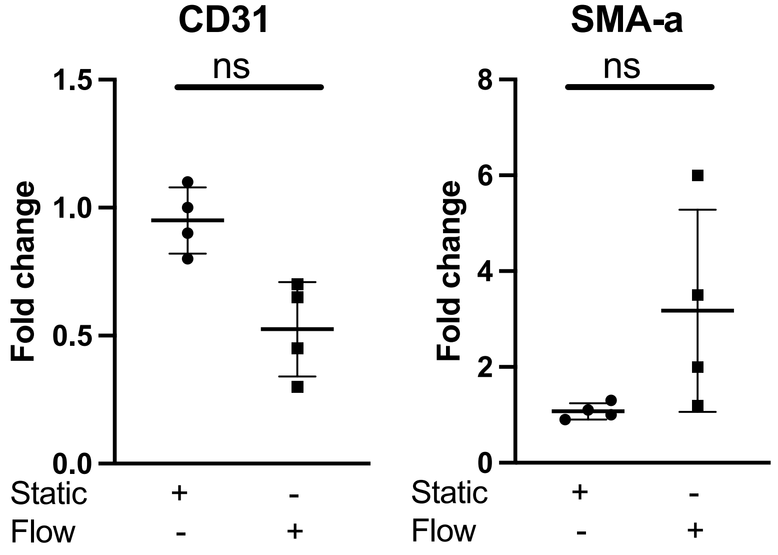

(B)

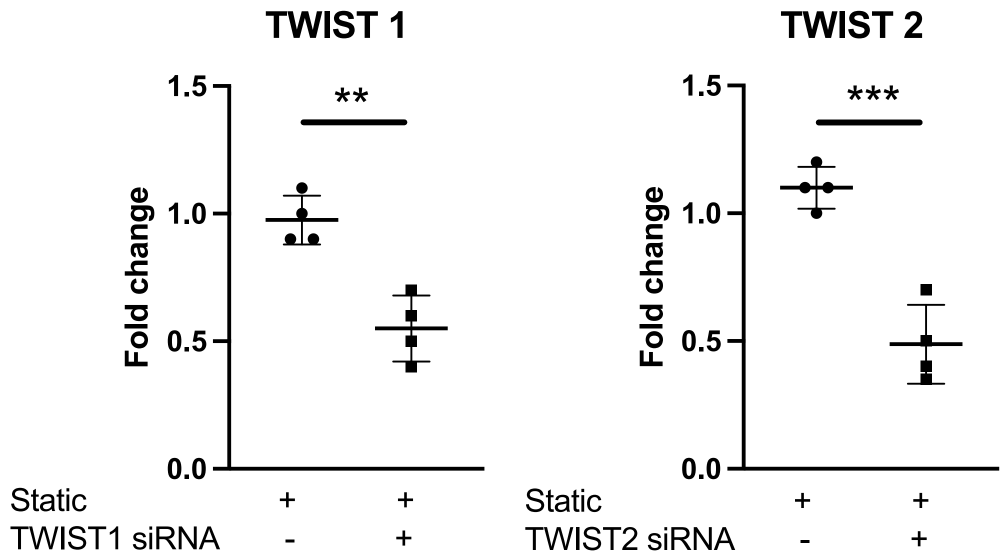

(C)

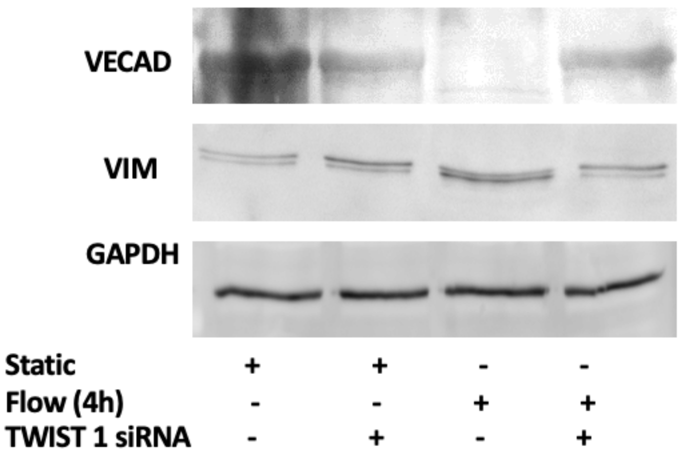
VECAD
VIM
GAPDH
Static + + - -
Flow (4h) - - + +
TWIST 1 siRNA - + - +

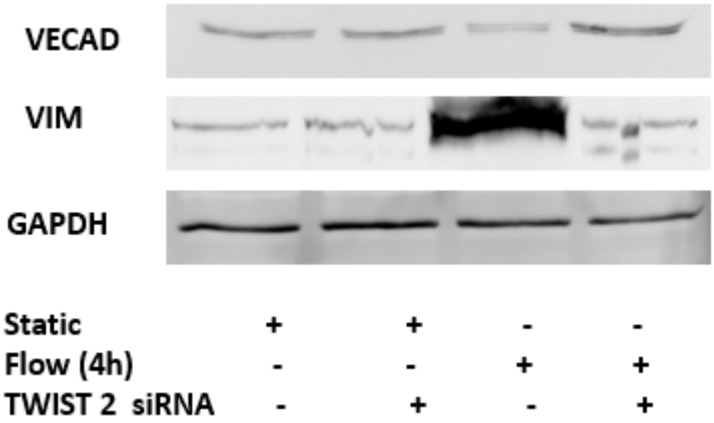
VECAD
VIM
GAPDH
Static + + - -
Flow (4h) - - + +
TWIST 2 siRNA - + - +

(D)

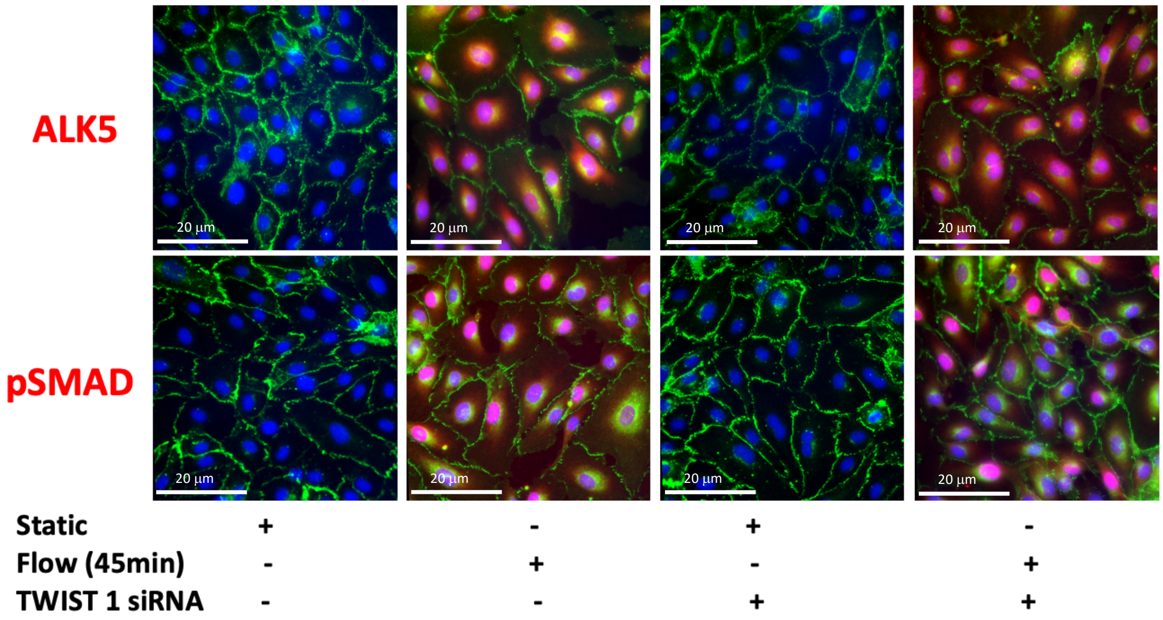
ALK5
pSMAD
20 µm
Static + - + -
Flow (45min) - + - +
TWIST 1 siRNA - - + +

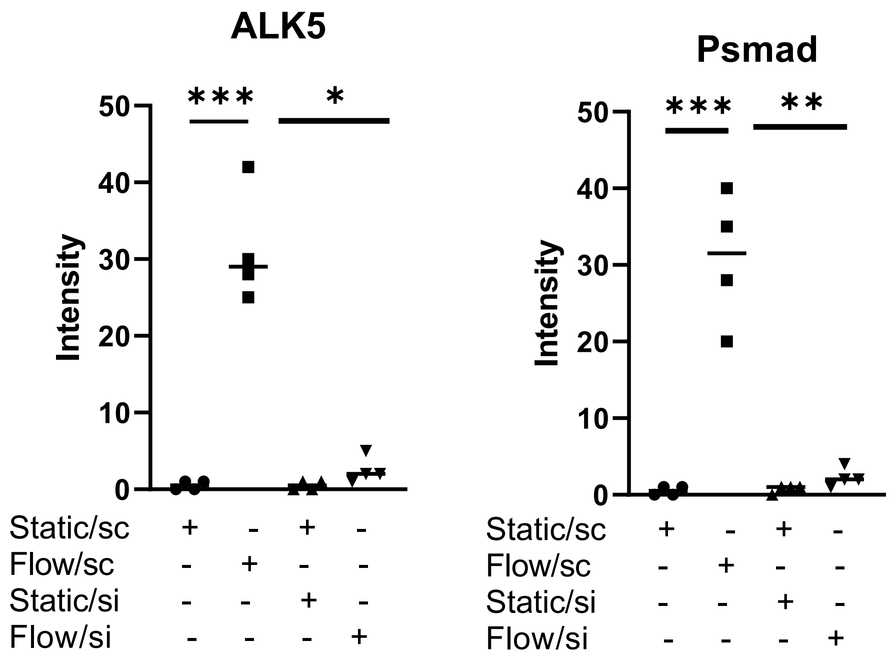
ALK5
Psmad
Intensity
***
*
**
0
10
20
30
40
50
Static/sc + - + -
Flow/sc - + - -
Static/si - - + -
Flow/si - - - +

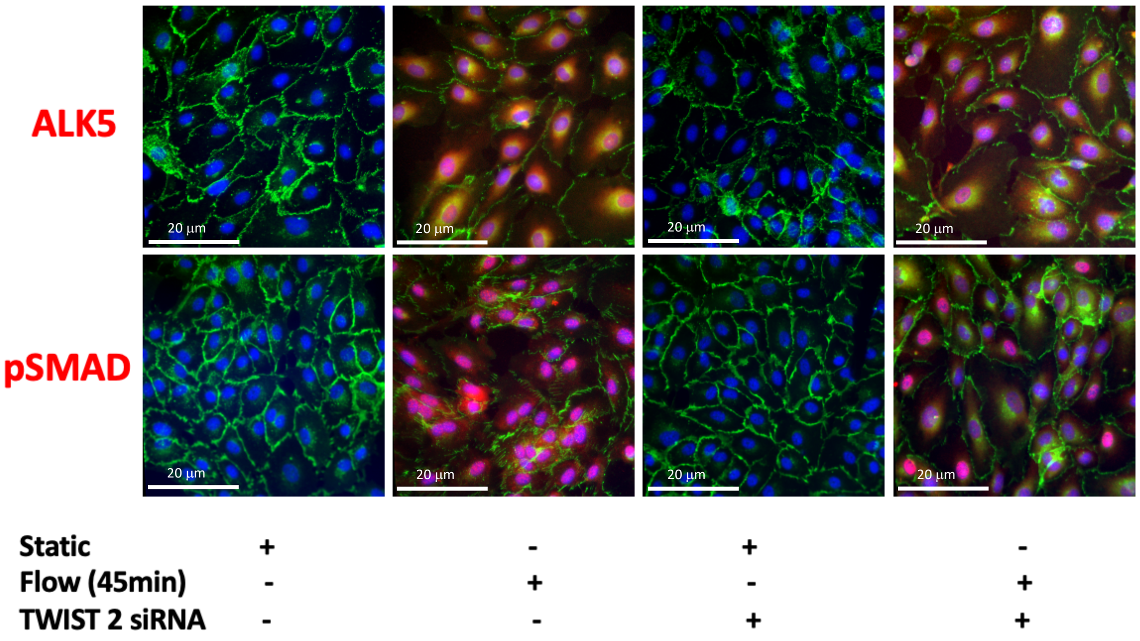
ALK5
pSMAD
20 µm
Static + - + -
Flow (45min) - + - +
TWIST 2 siRNA - - + +

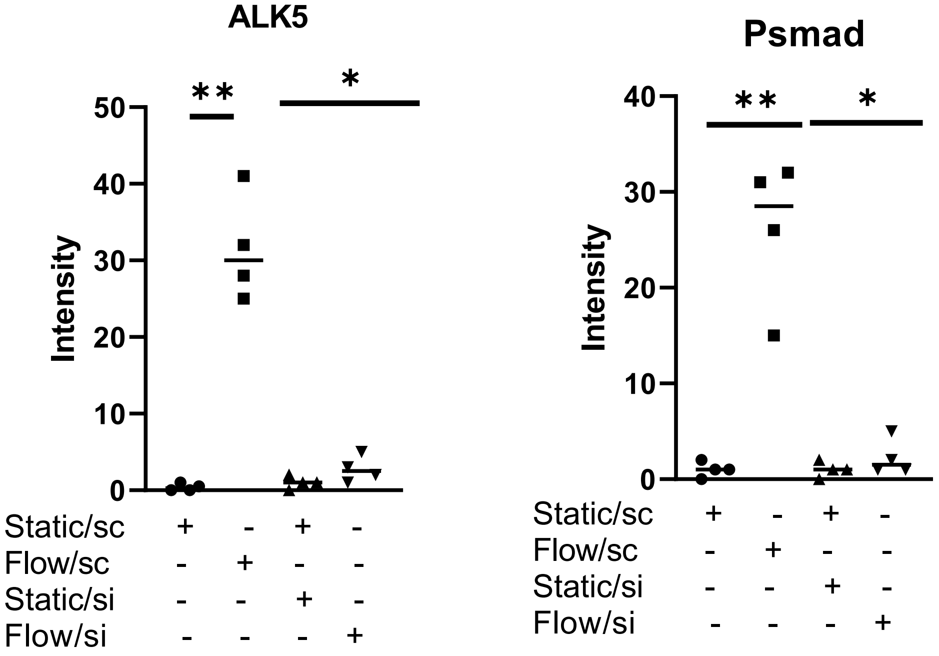

Supp. Figure S1. (A) HUVECs were cultured under static conditions or exposed to laminar shear stress (LSS) for 4 hours. The levels of CD31 and SMA transcripts were measured using comparative RT-PCR. The values are derived from four independent experiments. (B) HUVECs were transfected with either TWIST1 or TWIST2-specific siRNA or scrambled control (Scr). HUVECs were also cultured under static conditions or exposed to LSS for 4 hours, with transcript levels of TWIST1 and TWIST2 assessed through comparative RT-PCR. Again, values from four independent experiments are shown as mean values. (C) Representative Western blotting of total cell lysates of VIM, and VEcad assessed at 4 hours in HUVECs that were transfected with TWIST1 or TWIST2-specific siRNA or scrambled control (Scr), after exposure to LSS or static culture for 4 hours (see full gels). (D) HUVECs were transfected with TWIST1 or TWIST2-specific siRNA or a scrambled control (Scr). ells were then either exposed to LSS or cultured under static conditions.T he expression levels of ALK5 and P-SMAD 2/3 were evaluated at 45 minutes using immunofluorescence staining with specific antibodies. The results were quantified across multiple ECs. Representative images and values from four independent experiments are included.

Supp. Figure S2

(A)

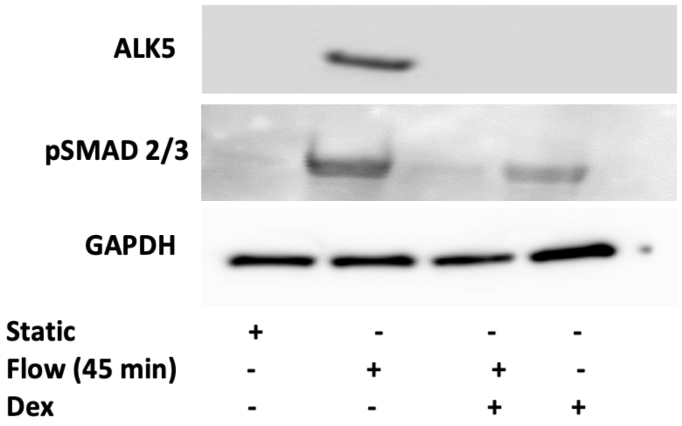

(B)

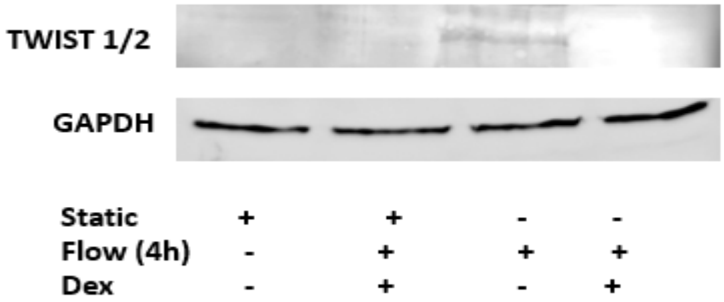

Supp. Figure S2. HUVECs were either pretreated with dexamethasone (10 µmol/L for 60 minutes) or left untreated. After treatment, they were cultured under static conditions or exposed to LSS for 45 minutes or 4 hours. Representative Western blotting of total cell lysates of ALK5 and pSMAD2/3 at 45 minutes (A) as well as TWIST1/2 at 4 hours gels).

Supp. Figure S3

(A)

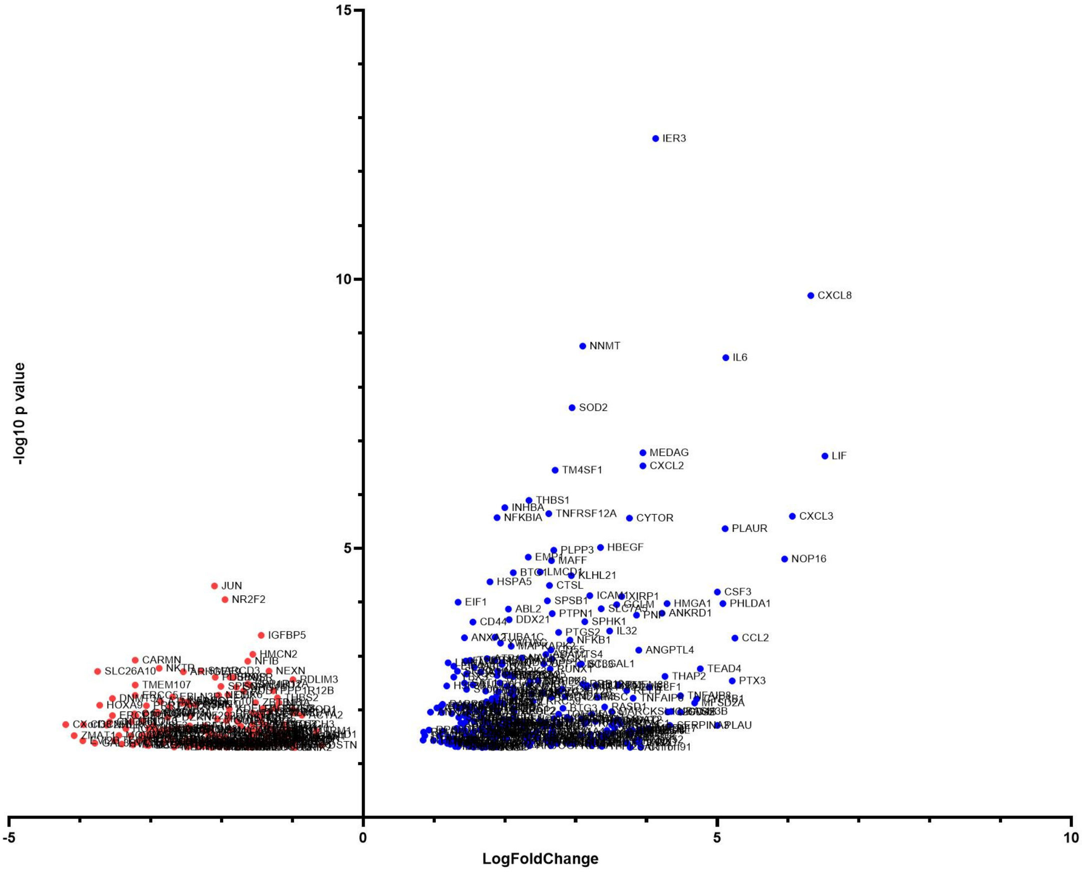

(B)

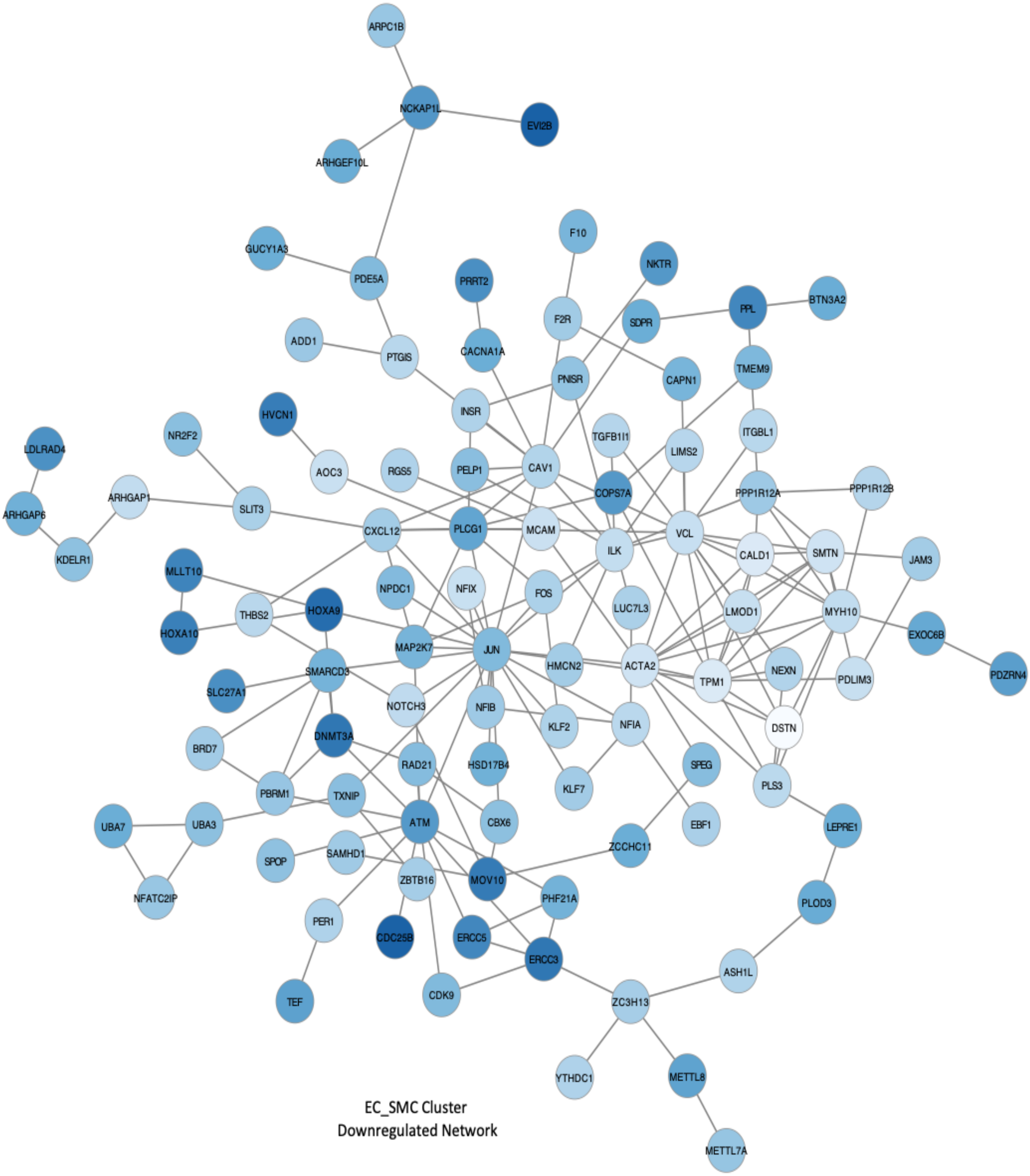

EC_SMC Cluster
Downregulated Network

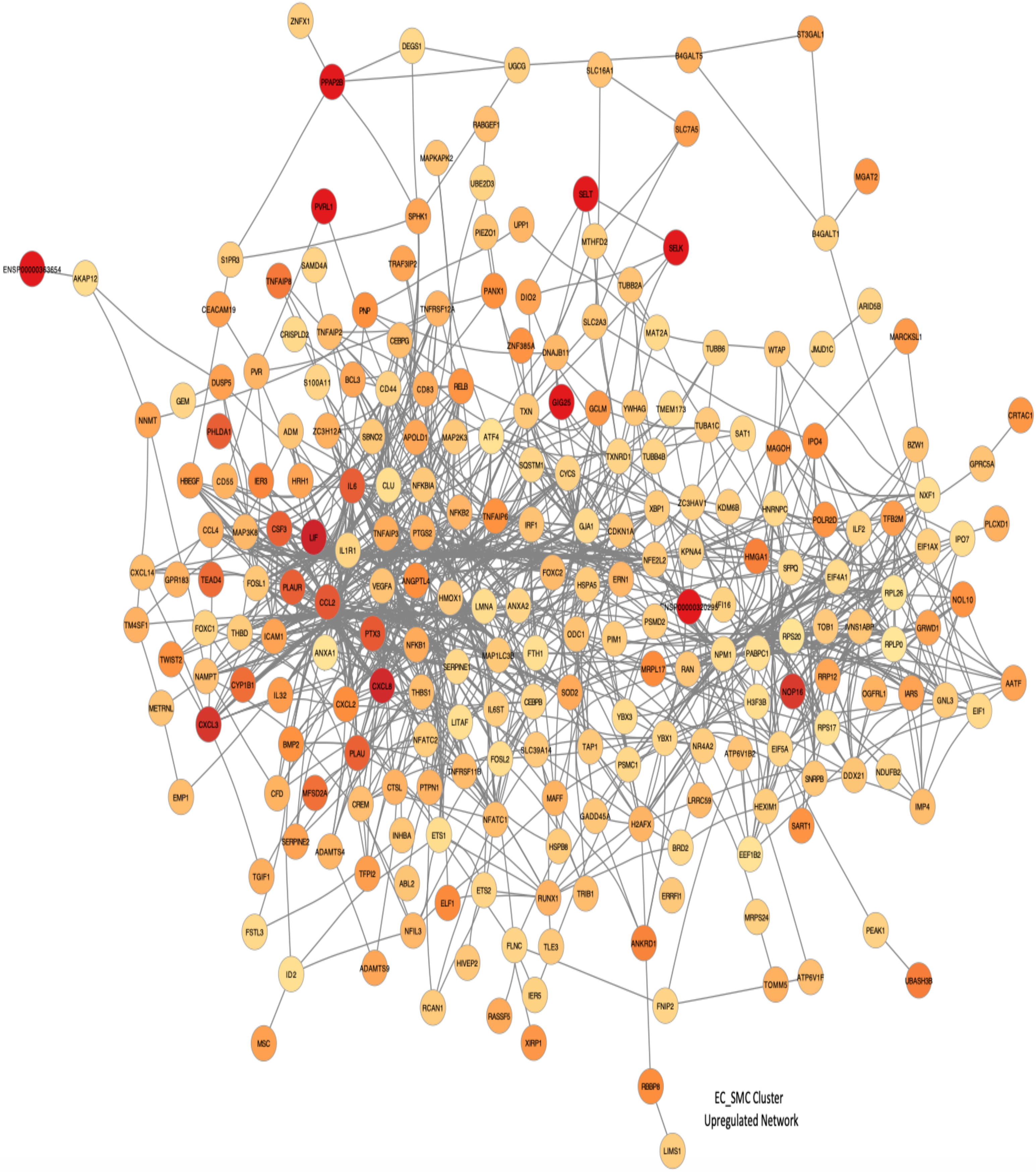

EC_SMC Cluster
Upregulated Network

Supp. Figure S3. (A)Volcano plot of the significant genes in the EndMT subcluster (B) Analysis of the EC/SMC cluster network identified all significantly differentially Supp Supp Figure.2.. expressed genes that were either upregulated or downregulated (adjusted p-value < 0.05) in samples exposed to ex vivo arterial hemodynamic conditions. The samples are color-coded on a red-gray gradient based on log fold change (logFC) values. Direct connections represent known interactions between these genes.

Supp. Figure S4
(A)

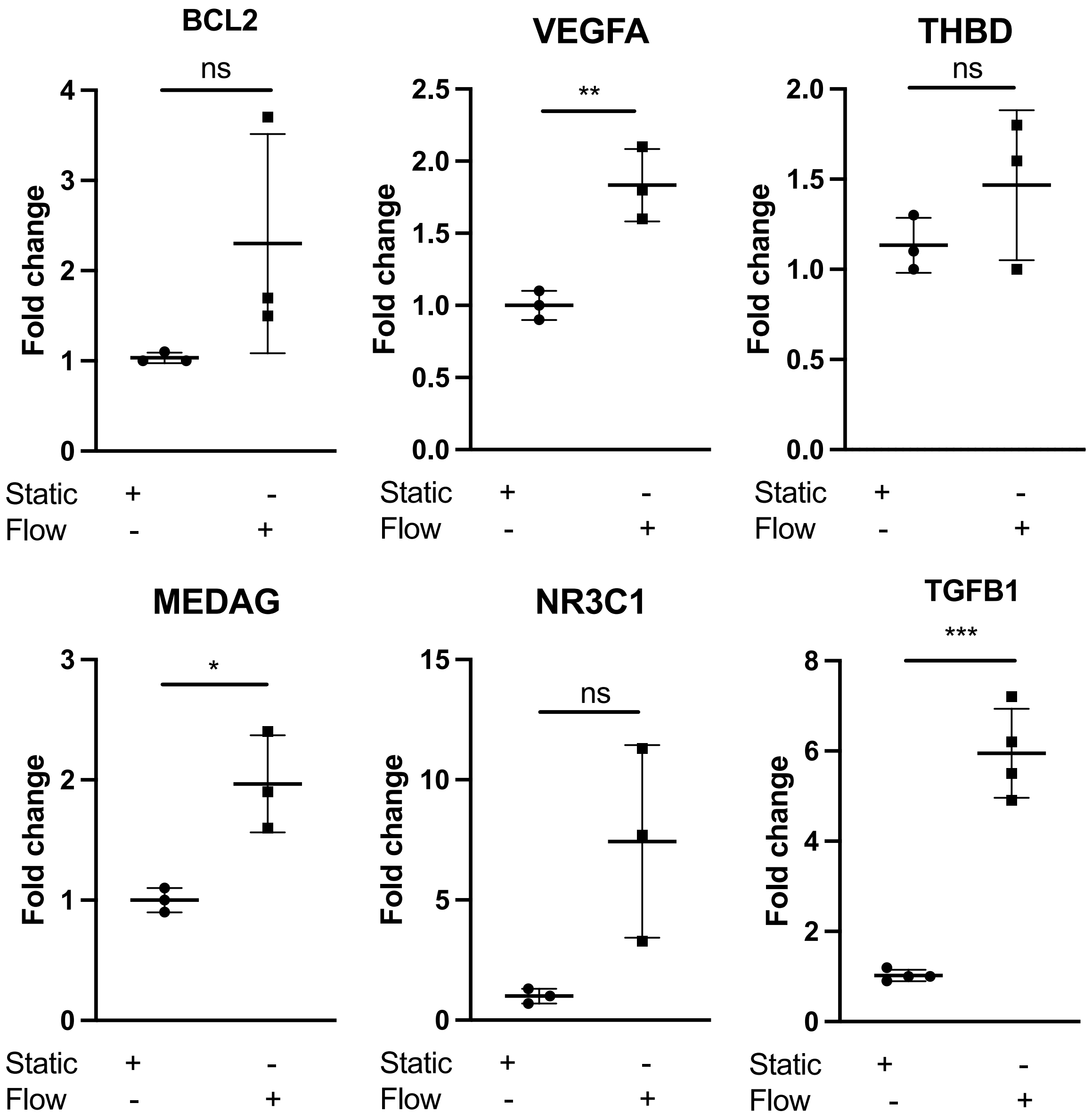

(B)

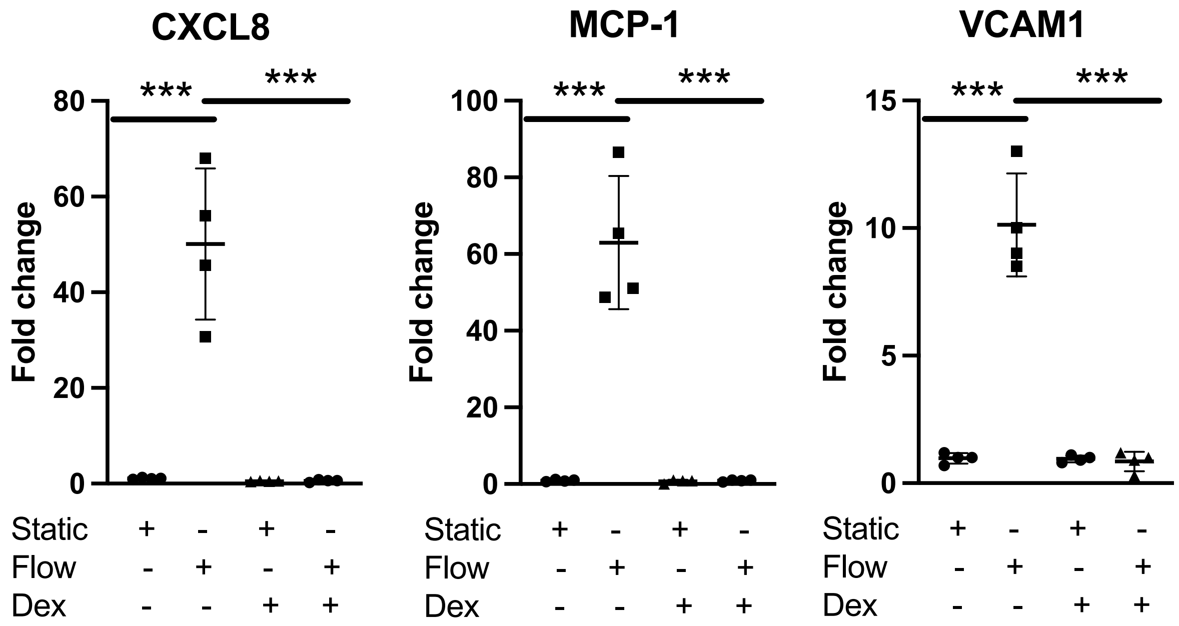
CXCL8
MCP-1
VCAM1
***
***
Fold change
0
20
40
60
80
100
5
10
15
Static + - + -
Flow - + - +
Dex - - + +

(C)

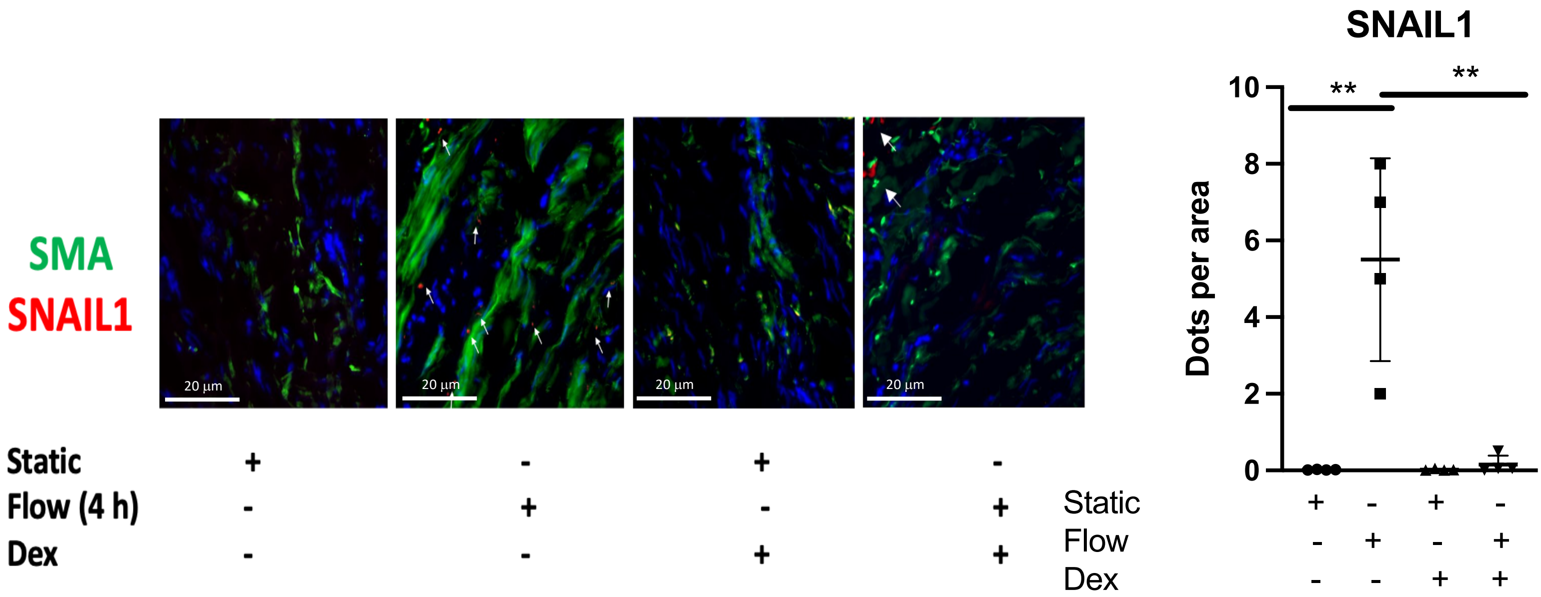
SMA
SNAIL1
20 µm
Static + - + -
Flow (4 h) - + - +
Dex - - + +
SNAIL1
**
**
Dots per area
0
2
4
6
8
10
Static + - + -
Flow - + - +
Dex - - + +

(D)

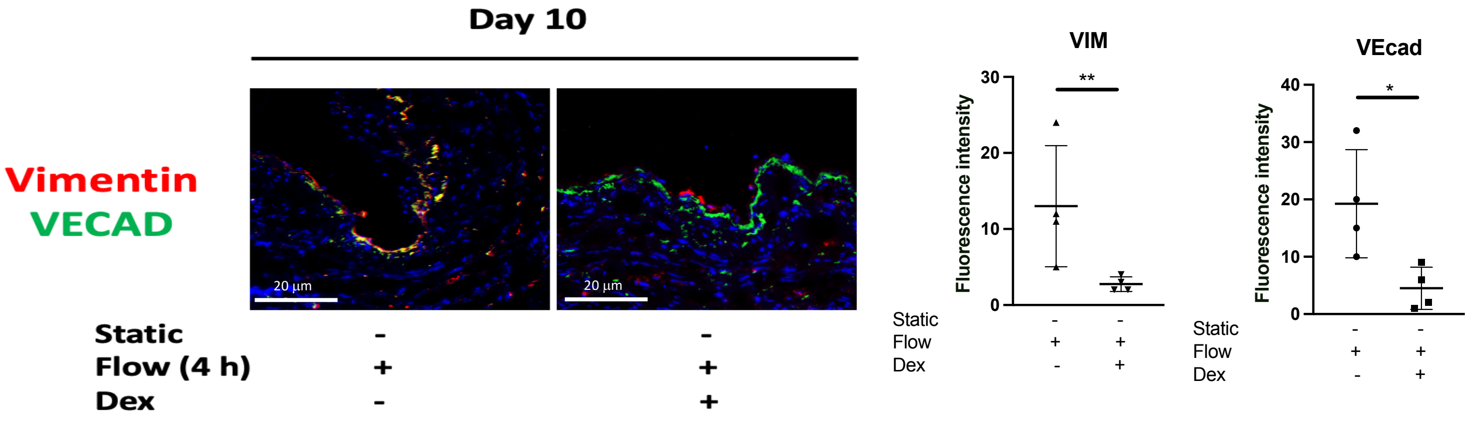

(E)

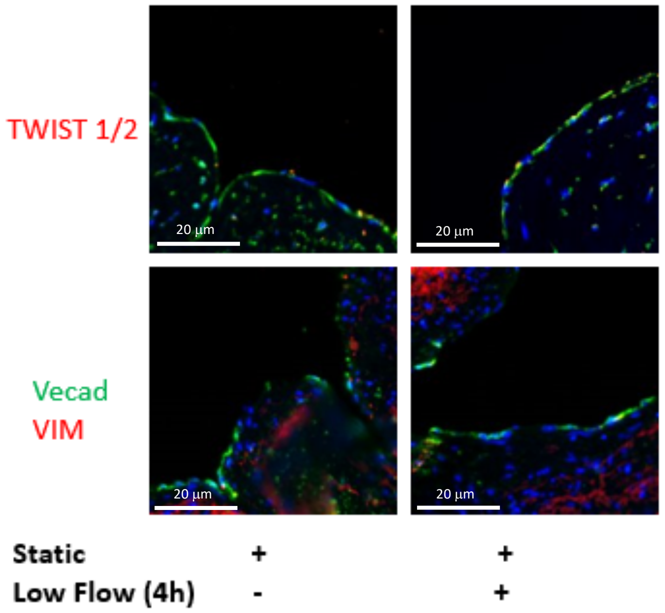

Supp. Figure S4(A) Validation of different other genes of interest under static or 4 hours arterial flow in LSV. (B) LSV were either pretreated with dexamethasone (10 µmol/L for 60 minutes) or left untreated. After treatment, they were mounted on a perfusion apparatus and exposed to LSS for 4 hours, or maintained under static conditions as a control. Comparative RT-PCR was then performed to measure the transcript levels of CXCL8, MCP-1, and VCAM1 in the whole tissue. Results from four independent experiments are presented. (C) Similarly, LSV were pretreated with dexamethasone (10 µmol/L for 60 minutes) or remained untreated. Afterward, they were mounted on a perfusion apparatus and subjected to arterial flow for 4 hours or kept under static conditions as a control. Comparative RT-PCR

subsequently validated the transcript levels of genes of interest derived from spatial sequencing data, with mean values shown from four independent experiments. Transcript expression levels of SNAIL1 at the 4-hour mark were assessed using RNAScope with probes specific for the SNAIL1 gene. The expression of SNAIL1 was quantified in multiple endothelial cells (ECs) and averaged for each experimental group, with representative images and data from four independent experiments provided. (D) LSV were either pretreated with dexamethasone (10 µmol/L for 60 minutes) or remained untreated. They were then mounted on a perfusion apparatus and exposed to arterial flow for 4 hours or maintained under static conditions as a control. The vein segments were subsequently cultured in a well for 10 days. The expression levels of VIM and VEcad were assessed using immunofluorescence staining with specific antibodies. These levels were quantified in multiple ECs with representative images and results from four independent experiments presented. (E) LSV were either mounted on a perfusion apparatus and exposed to low shear stress for 4 hours or maintained under static conditions as a control. The expression levels of TWIST1/2, VIM, and VEcad were evaluated at the 4 hour using immunofluorescence staining with specific antibodies.

Supplementary table S1. Antibodies used in the study

**Antibodies**	**Manufacturer**	**Product code**
Anti- VE-CADHERIN	Invitrogen	14-1449-82
Anti- PECAM1	R&D Systems	BBA7
Anti-VIMENTIN	Cell Signaling Technology	5741
Anti-αSMA	Antibodies.com	A82445
Anti-ALK5 (TGFBR1)	Antibodies.com	A256954-200
Anti-Phospho-p38 MAPK	Cell Signaling Technology	4511
Anti-Phospho-SMAD2/3	R&D Systems	MAB8935
Anti-TWIST1/2	Abcam	ab50887
Anti-GAPDH	ThermoFisher Scientific	MA5-15738
anti-Mouse Alexa Fluor™ 488	ThermoFisher Scientific	A-11001
anti-Goat Alexa Fluor™ 488	ThermoFisher Scientific	A-11078
anti-Rabbit, Alexa Fluor™ 488	ThermoFisher Scientific	A-11008
anti-Mouse Alexa Fluor™ 568	ThermoFisher Scientific	A11031
anti-Goat Alexa Fluor™ 594	ThermoFisher Scientific	A11058
anti-Rabbit, Alexa Fluor™ 568	ThermoFisher Scientific	A11011
Anti-mouse IgG, HRP-linked	Cell Signaling Technology	7076S
Anti-goat IgG, HRP-linked	Santa Cruz Biotechnology	sc-2354
Anti-rabbit IgG, HRP-linked	Cell Signaling Technology	7074

Supplementary table S2. Primers list

**Primers**	**Manufacturer**	**Assay ID**
CD31	ThermoFisher Scientific	Hs00169777_m1
VE-CADHERIN	ThermoFisher Scientific	Hs00901465_m1
ACTA2	ThermoFisher Scientific	Hs00909449_m1
PPIA	ThermoFisher Scientific	Hs99999904_m1
TWIST1	ThermoFisher Scientific	Hs00361186_m1
TWIST2	ThermoFisher Scientific	Hs00382379_m1
TGFβ 1	ThermoFisher Scientific	Hs00998133_m1
CCL2	ThermoFisher Scientific	Hs00234140_m1
IL-8	ThermoFisher Scientific	Hs00174103_m1
SNAI1	ThermoFisher Scientific	Hs00195591_m1
FOXC1	ThermoFisher Scientific	Hs00559473_s1
FOXC2	ThermoFisher Scientific	Hs00270951_s1
REL	ThermoFisher Scientific	Hs00968440_m1
TG1F1	ThermoFisher Scientific	Hs07289533_m1
CD44	ThermoFisher Scientific	Hs01075864_m1

Supplementary table S3. Significantly regulated genes in the cluster subset

gene	logFC	lfcSE	P
**IER3**	4.13	0.563	2.41E-13
**CXCL8**	6.32	0.993	2E-10
**NNMT**	3.1	0.514	1.74E-09
**IL6**	5.12	0.862	2.85E-09
**SOD2**	2.95	0.529	2.43E-08
**MEDAG**	3.95	0.756	1.67E-07
**LIF**	6.52	1.25	1.91E-07
**CXCL2**	3.95	0.769	2.91E-07
**TM4SF1**	2.71	0.532	3.5E-07
**THBS1**	2.34	0.483	1.28E-06
**INHBA**	2	0.418	1.74E-06
**TNFRSF12A**	2.62	0.554	2.26E-06
**CXCL3**	6.06	1.29	2.53E-06
**NFKBIA**	1.89	0.403	2.67E-06
**CYTOR**	3.76	0.801	2.73E-06
**PLAUR**	5.11	1.11	4.3E-06
**HBEGF**	3.35	0.756	9.63E-06
**PLPP3**	2.69	0.612	1.08E-05
**EMP1**	2.33	0.537	1.45E-05
**NOP16**	5.95	1.38	1.57E-05
**MAFF**	2.66	0.619	1.68E-05
**LMCD1**	2.5	0.596	2.75E-05
**BTG1**	2.12	0.506	2.83E-05
**KLHL21**	2.94	0.707	3.2E-05
**HSPA5**	1.79	0.437	4.18E-05
**CTSL**	2.63	0.648	4.89E-05
**JUN**	-2.1	0.518	4.98E-05
**CSF3**	5	1.25	6.47E-05
**ICAM1**	3.2	0.808	7.55E-05
**XIRP1**	3.65	0.924	7.82E-05
**NR2F2**	-1.95	0.497	8.92E-05
**SPSB1**	2.6	0.666	9.41E-05
**EIF1**	1.34	0.343	9.96E-05
**HMGA1**	4.29	1.11	0.000106
**PHLDA1**	5.08	1.31	0.000106
**GCLM**	3.58	0.926	0.000111
**SLC7A5**	3.36	0.878	0.000132
**ABL2**	2.05	0.536	0.000134
**ANKRD1**	4.22	1.12	0.00016

**PTPN1**	2.67	0.708	0.000163
**PNP**	3.86	1.03	0.000173
**DDX21**	2.06	0.555	0.00021
**SPHK1**	3.13	0.85	0.00023
**CD44**	1.55	0.421	0.000233
**IL32**	3.48	0.971	0.000343
**PTGS2**	2.76	0.774	0.000364
**IGFBP5**	-1.44	0.408	0.000411
**TUBA1C**	1.86	0.53	0.000446
**ANXA2**	1.43	0.409	0.000457
**CCL2**	5.25	1.5	0.000465
**NFKB1**	2.92	0.84	0.000506
**YWHAG**	1.94	0.562	0.000576
**MAPKAPK2**	2.09	0.612	0.000654
**CD55**	2.65	0.787	0.000773
**ANGPTL4**	3.89	1.16	0.000777
**HMCN2**	-1.56	0.472	0.000929
**ADAMTS4**	2.58	0.779	0.000934
**NAMPT**	2.24	0.685	0.00108
**ATP13A3**	1.75	0.539	0.00112
**SGK1**	2.67	0.82	0.00113
**CARMN**	-3.22	0.993	0.00119
**TUBB4B**	1.51	0.466	0.0012
**SLC39A14**	1.86	0.574	0.00121
**FLNC**	1.45	0.448	0.00124
**NFIB**	-1.63	0.506	0.00125
**GADD45A**	1.96	0.611	0.00133
**LMNA**	1.2	0.374	0.00133
**UPP1**	2.55	0.798	0.00139
**ST3GAL1**	3.08	0.963	0.0014
**RAN**	1.96	0.615	0.00142
**BCL3**	3.06	0.96	0.00143
**EIF4A1**	1.28	0.403	0.00155
**UBE2D3**	1.57	0.496	0.0016
**NKTR**	-2.88	0.917	0.00169
**TEAD4**	4.76	1.52	0.00172
**RUNX1**	2.64	0.844	0.00175
**SMARCD3**	-2.29	0.737	0.00188
**NEXN**	-1.33	0.43	0.0019
**SFPQ**	1.33	0.429	0.00192
**MAP2K3**	1.9	0.611	0.00192
**SLC26A10**	-3.75	1.21	0.00193

**ARHGEF9**	-2.54	0.82	0.00196
**ATP1B3**	1.66	0.536	0.00199
**SQSTM1**	1.46	0.474	0.00215
**SLC25A25**	2.04	0.668	0.00222
**SLC2A3**	2	0.654	0.00222
**ERRFI1**	1.89	0.619	0.00231
**TUBB2A**	2.08	0.681	0.00231
**THAP2**	4.26	1.4	0.00238
**METRNL**	2.12	0.7	0.0024
**YBX3**	1.28	0.421	0.00246
**PDE5A**	-2.09	0.691	0.00248
**SPOP**	-1.88	0.622	0.00248
**PNISR**	-1.83	0.604	0.00249
**PDLIM3**	-0.993	0.332	0.00275
**MAP3K8**	2.47	0.829	0.00289
**PTX3**	5.21	1.75	0.0029
**TNFAIP2**	2.36	0.792	0.00295
**WTAP**	1.93	0.654	0.00316
**SAT1**	1.43	0.486	0.00319
**PPP1R12A**	-1.63	0.554	0.00332
**RRP12**	3.1	1.06	0.00334
**TRIB1**	2.34	0.797	0.00336
**TUBB6**	1.55	0.53	0.00342
**SAMHD1**	-1.6	0.546	0.00345
**TMEM88**	3.61	1.24	0.00346
**TMEM107**	-3.22	1.1	0.00346
**DUSP5**	3.28	1.12	0.00351
**SELENOT**	3.16	1.08	0.00354
**H3F3B**	1.18	0.407	0.00358
**CDKN1A**	2.03	0.699	0.00363
**SPEG**	-2.01	0.691	0.00367
**ELF1**	4.04	1.39	0.0038
**SNHG14**	-1.79	0.618	0.0038
**MIR4435-2HG**	3.15	1.09	0.00381
**TSC22D2**	2.12	0.736	0.00396
**TGIF1**	2.81	0.981	0.00413
**S100A11**	1.46	0.508	0.00415
**UBL3**	2.12	0.74	0.00417
**RELB**	3.72	1.31	0.00439
**PPP1R12B**	-1.27	0.447	0.0044
**ADD1**	-1.69	0.593	0.00443

**IL6ST**	1.73	0.609	0.00447
**ZC3H12A**	2.76	0.975	0.00457
**MAP1LC3B**	1.91	0.674	0.0046
**NFIL3**	2.54	0.9	0.00481
**VEGFA**	2.09	0.744	0.00492
**GPRC5A**	2.08	0.74	0.00492
**FOXC2**	2.38	0.848	0.00502
**PIEZO1**	2.12	0.757	0.00514
**CBX6**	-1.91	0.686	0.00528
**NEMF**	-2.04	0.732	0.00533
**ERCC5**	-3.22	1.16	0.0054
**TNFAIP8**	4.48	1.61	0.00547
**ELOVL5**	1.88	0.678	0.00566
**TNFAIP3**	2.85	1.03	0.00568
**EBLN3P**	-2.69	0.973	0.00574
**MSC**	3.31	1.2	0.00585
**CDC42EP4**	2.66	0.964	0.00585
**BZW1**	2.13	0.773	0.00586
**THBS2**	-1.21	0.441	0.00595
**TNFAIP6**	3.81	1.39	0.00607
**DNMT3A**	-3.54	1.29	0.00615
**PIM1**	1.82	0.663	0.00618
**CYP1B1**	4.71	1.72	0.00627
**THBD**	1.83	0.674	0.0067
**NFASC**	-2.56	0.943	0.00675
**LRRC59**	2.4	0.889	0.00694
**ARHGEF10L**	-2.47	0.917	0.00695
**ATM**	-2.86	1.06	0.00708
**HEXIM1**	1.74	0.646	0.00709
**NFE2L2**	1.89	0.704	0.00716
**ZBTB16**	-1.52	0.568	0.00733
**NFIA**	-1.19	0.445	0.0074
**FOSL1**	1.95	0.73	0.00741
**CABIN1**	-2.55	0.952	0.00744
**MFSD2A**	4.68	1.75	0.00745
**RND3**	1.98	0.743	0.00763
**PABPC1**	1.12	0.422	0.00792
**RSBN1L**	-2.36	0.888	0.00795
**ZC3H6**	-2.45	0.924	0.00812
**HOXA9**	-3.72	1.41	0.00819
**MAT2A**	1.3	0.492	0.00826
**PRRT2**	-3.06	1.16	0.00839

**YBX1**	1.42	0.539	0.00846
**RPS17**	1.1	0.417	0.00854
**SAMD4A**	1.51	0.574	0.00869
**MIR22HG**	1.68	0.641	0.00876
**FAM207A**	-2.73	1.04	0.00878
**RASD1**	3.41	1.3	0.00884
**HSPB8**	1.84	0.703	0.0089
**ATF4**	1.04	0.399	0.00925
**FOS**	-1.41	0.544	0.00933
**HNRNPC**	1.35	0.521	0.00939
**BTG3**	2.82	1.08	0.00939
**LIMS2**	-1.24	0.478	0.0094
**ODC1**	2.12	0.817	0.00949
**PTGIS**	-1.22	0.472	0.00952
**KDELR1**	-1.89	0.731	0.00959
**LMOD1**	-0.99	0.383	0.00984
**PALM2-AKAP2**	1.6	0.624	0.0104
**TXN**	1.89	0.739	0.0105
**APOL2**	2.13	0.835	0.0106
**CAV1**	-1.32	0.518	0.0107
**UBASH3B**	4.35	1.7	0.0107
**MARCKSL1**	3.51	1.38	0.0108
**C2CD4B**	4.3	1.69	0.0109
**G0S2**	4.48	1.76	0.011
**ANXA1**	0.951	0.374	0.011
**ETS2**	1.49	0.587	0.011
**LIMS1**	1.7	0.669	0.0111
**LUC7L3**	-1.43	0.563	0.0111
**FTH1**	1.11	0.438	0.0112
**PWWP3A**	-2.9	1.14	0.0112
**MCAM**	-0.944	0.372	0.0113
**CXCL12**	-1.56	0.617	0.0114
**VCL**	-0.979	0.387	0.0114
**FAM76A**	-3.07	1.22	0.0115
**AKAP12**	1.25	0.494	0.0117
**NCKAP1L**	-2.92	1.16	0.0117
**PRMT2**	-1.9	0.756	0.0118
**C1orf35**	-3.23	1.29	0.0119
**TOMM5**	2.76	1.1	0.012
**IARS**	3.23	1.29	0.0121
**RRAS**	-1.51	0.602	0.0123

**ACTA2**	-0.865	0.347	0.0126
**ERCC3**	-3.54	1.42	0.0128
**FNIP2**	1.43	0.576	0.0128
**ZNF292**	-2.44	0.982	0.013
**PPL**	-3.21	1.29	0.013
**B4GALT1**	1.66	0.673	0.0136
**CD83**	2.88	1.17	0.0137
**PDZRN4**	-2.76	1.12	0.0137
**EBF1**	-1.45	0.588	0.0138
**SERPINE1**	1.35	0.547	0.0138
**IRF1**	2.12	0.862	0.014
**WASHC1**	-1.89	0.771	0.014
**B4GALT5**	2.69	1.1	0.0146
**ADAMTS9**	3.08	1.26	0.0147
**JMJD1C**	1.49	0.61	0.0147
**MKLN1**	-2.06	0.845	0.0148
**SELENOK**	1.97	0.81	0.015
**VPS37B**	2.93	1.21	0.015
**MGAT2**	3.61	1.49	0.0154
**GUCY1A1**	-1.93	0.8	0.0159
**BAIAP2**	3.56	1.48	0.0159
**ERN1.00**	2.49	1.03	0.016
**NINJ1**	2.06	0.857	0.0161
**ARID5B**	1.46	0.609	0.0162
**NOL3**	-3.11	1.3	0.0162
**HRH1**	3.16	1.31	0.0163
**COQ10B**	1.71	0.711	0.0164
**PHLDA2**	3.28	1.37	0.0164
**IFI16**	1.57	0.656	0.0165
**MLLT10**	-3.27	1.37	0.0166
**METTL7A**	-1.77	0.739	0.0166
**CNOT6L**	-3.58	1.5	0.0168
**SLIT3**	-1.44	0.603	0.0168
**PSMD2**	1.76	0.738	0.0171
**CITED2**	2.58	1.08	0.0173
**CDC42EP3**	1.38	0.58	0.0175
**CREM**	2.25	0.947	0.0176
**ATP6V1B2**	2.07	0.873	0.0178
**TRAF3IP2**	3.13	1.32	0.0179
**GEM**	1.42	0.602	0.0179
**CRTAC1**	3.63	1.54	0.0181
**SLC16A1**	2.16	0.913	0.0182

**CCL4**	2.77	1.18	0.0184
**AL627171.2**	-1.56	0.665	0.0186
**NOTCH3**	-1.12	0.475	0.0186
**CDC25B**	-3.95	1.68	0.0188
**CXorf38**	-4.2	1.79	0.0188
**SBNO2**	1.92	0.819	0.0189
**MAGOH**	3.51	1.5	0.0191
**NHLRC3**	-3.62	1.54	0.0191
**KLF2**	-1.38	0.587	0.0191
**PLAU**	5	2.14	0.0195
**SERPINA3**	4.33	1.85	0.0195
**USF3**	-2.96	1.27	0.0196
**RABGEF1**	2.27	0.972	0.0197
**UBA7**	-2.45	1.05	0.0199
**F10**	-2.23	0.957	0.0199
**CD302**	-1.73	0.743	0.02
**YTHDC1**	-1.36	0.583	0.02
**SNRPB**	2.02	0.871	0.0202
**CFD**	2.66	1.15	0.0204
**NFKB2**	2.56	1.11	0.0206
**HIVEP2**	1.96	0.847	0.0207
**ZC3HAV1**	1.68	0.726	0.0207
**RCAN1**	1.81	0.787	0.0212
**ATP6V1F**	2.26	0.987	0.0218
**AC090617.10**	3.55	1.55	0.0218
**CRISPLD2**	1.32	0.578	0.022
**CHKB**	-3.03	1.32	0.0221
**FTX**	-2.5	1.09	0.0221
**MRPL17**	4.03	1.76	0.0222
**HOXA10**	-3.35	1.47	0.0223
**MYH10**	-1.08	0.472	0.0223
**ASB8**	-2.62	1.15	0.0224
**ANKHD1**	-1.29	0.564	0.0226
**PHLDA3**	-2.46	1.08	0.0229
**GOLGA6L4**	-2.73	1.2	0.0233
**ILK**	-1.05	0.462	0.0234
**MICAL1**	-1.87	0.824	0.0235
**DUT**	-1.62	0.714	0.0235
**TBC1D1**	-1.48	0.656	0.0236
**EIF1AX**	1.65	0.727	0.0236
**PTPN18**	-2.84	1.26	0.0237
**CEBPB**	1.43	0.632	0.0237

**RPS20**	0.928	0.411	0.0238
**LGI4**	-2.15	0.95	0.0238
**TMEM173**	1.35	0.599	0.0239
**BMP2**	3.8	1.68	0.0239
**RUBCNL**	3.9	1.73	0.024
**STX11**	3.44	1.52	0.0241
**P3H1**	-2.75	1.22	0.0243
**RBBP8**	3.95	1.75	0.0243
**TMEM80**	-2.94	1.31	0.0244
**ZCRB1**	-1.56	0.694	0.0245
**ZNF385A**	3.76	1.67	0.0246
**CAPN1**	-2.24	0.999	0.0247
**TPM1**	-0.662	0.295	0.0247
**CLU**	1.13	0.505	0.0248
**FAM180A**	2.45	1.09	0.025
**TGFB1I1**	-1.27	0.567	0.025
**PLS3**	-1.17	0.524	0.0252
**SLC41A1**	2.68	1.2	0.0254
**MTHFD2**	1.74	0.778	0.0256
**CYCS**	1.56	0.698	0.0257
**RPLP0**	0.853	0.383	0.0259
**STK17A**	1.89	0.848	0.026
**ITPRIP**	2.11	0.948	0.0263
**NOL10**	3.75	1.69	0.0264
**COPS7A**	-2.88	1.3	0.0264
**GNL3**	1.85	0.834	0.0266
**OAF**	2.66	1.2	0.0266
**MTERF4**	-2.14	0.968	0.0268
**S1PR3**	1.76	0.793	0.0268
**CDC42EP2**	3.52	1.59	0.0269
**TAP1**	1.89	0.856	0.0272
**TENT5A**	1.72	0.781	0.0272
**ZC3H13**	-1.51	0.684	0.0273
**TWIST2**	3.78	1.72	0.0282
**CHST12**	-1.54	0.702	0.0284
**AC007952.4**	-3.03	1.38	0.0284
**SERPINE2**	3.21	1.47	0.0286
**CALD1**	-0.665	0.304	0.0287
**ASH1L**	-1.35	0.616	0.0289
**TXNRD1**	1.45	0.662	0.0289
**KDM6B**	1.73	0.791	0.0289
**RNF19B**	1.91	0.876	0.029

**KPNA4**	1.54	0.705	0.029
**BLOC1S6**	2.39	1.1	0.029
**KLF7**	-1.58	0.725	0.0291
**MT1F**	-2.93	1.35	0.0293
**CEBPG**	2.32	1.07	0.0295
**ZNF670**	3.35	1.54	0.0297
**LUCAT1**	3.42	1.57	0.0297
**APOLD1**	3.07	1.41	0.0297
**MOV10**	-3.45	1.59	0.0298
**RBFA**	-3.12	1.44	0.0299
**ECRG4**	-2.18	1	0.03
**MBNL1-AS1**	-1.2	0.553	0.03
**CDK9**	-2.1	0.967	0.03
**LRRC23**	-2.75	1.27	0.03
**RPL26**	0.87	0.401	0.03
**ZMAT1**	-4.08	1.88	0.03
**PER1**	-1.36	0.625	0.0301
**GLI4**	-3.28	1.51	0.0302
**ID2**	1.08	0.5	0.0303
**H2AFX**	2.61	1.21	0.0307
**INSR**	-1.37	0.633	0.0309
**ARHGAP1**	-1.06	0.494	0.0311
**NKAP**	-3.01	1.4	0.0311
**CCNDBP1**	-2.02	0.939	0.0312
**NFATC2**	1.78	0.827	0.0314
**MIR222HG**	2.69	1.25	0.0314
**OSBPL9**	-1.56	0.728	0.0316
**FOSL2**	1.2	0.557	0.0318
**TFB2M**	3.42	1.6	0.0323
**DNAJB11**	2.45	1.15	0.0325
**ZBTB7A**	-1.1	0.517	0.0325
**CEACAM19**	3.37	1.58	0.0325
**MAP7D3**	-1.94	0.905	0.0325
**IVNS1ABP**	2	0.937	0.033
**FOXC1**	1.57	0.735	0.033
**SMTN**	-0.854	0.402	0.0335
**CXCL14**	2.31	1.09	0.0336
**NPM1**	1.28	0.602	0.0338
**HCG11**	-3.13	1.47	0.0338
**JAM3**	-1.56	0.734	0.0338
**TXNIP**	-1.96	0.925	0.034
**NUCB1**	-1.15	0.542	0.034

**RGS5**	-1.36	0.641	0.0341
**ADM**	2.03	0.961	0.0346
**AOC3**	-0.95	0.45	0.0349
**F2R**	-1.59	0.753	0.0351
**TOB1**	1.86	0.883	0.0351
**LDLRAD4**	-3.03	1.44	0.0351
**PHYHIP**	-2.7	1.28	0.0353
**DIO2**	3.21	1.53	0.0356
**C3orf52**	3.89	1.86	0.0359
**FAM184B**	-1.2	0.571	0.036
**PLOD3**	-2.49	1.19	0.0361
**MAGI2-AS3**	-1.37	0.652	0.0362
**MXD1**	2.59	1.24	0.0364
**MTRNR2L12**	0.845	0.404	0.0364
**WDR11**	-2.42	1.16	0.0368
**TEF**	-2.7	1.29	0.0368
**TECPR1**	-3.51	1.68	0.0369
**EEF1B2**	0.992	0.476	0.0371
**ILF2**	1.53	0.735	0.0372
**BRD2**	1.35	0.649	0.0374
**GPR183**	2.67	1.28	0.0375
**EVI2B**	-3.96	1.91	0.0376
**SP3**	-2.22	1.07	0.0378
**MAP7D1**	1.27	0.612	0.038
**IMP4**	2.33	1.13	0.038
**PANX1**	3.85	1.86	0.038
**ETS1**	1.18	0.568	0.038
**MAST2**	-1.76	0.849	0.0383
**PVR**	2.6	1.25	0.0383
**NFATC2IP**	-1.74	0.842	0.0386
**PELP1**	-1.96	0.949	0.0387
**TACC1**	-0.982	0.476	0.0389
**LITAF**	1.1	0.534	0.0391
**TUBB3**	3.26	1.58	0.0391
**KBTBD2**	2.88	1.4	0.0393
**AC090114.2**	-2.81	1.37	0.0395
**NPDC1**	-2.06	1	0.0395
**C12orf75**	-1.25	0.606	0.0395
**L3HYPDH**	-3.19	1.55	0.0398
**EXOC6B**	-2.54	1.24	0.04
**TNFRSF11B**	2.37	1.16	0.0401
**R3HDM2**	-2.2	1.08	0.0405

**STX16**	-1.99	0.973	0.0407
**POLR2D**	3.72	1.82	0.0409
**IPO4**	3.9	1.91	0.041
**GRWD1**	3.18	1.56	0.0411
**FAM193B**	-2.83	1.39	0.0413
**NFATC1**	2.31	1.13	0.0414
**TUT4**	-3.06	1.5	0.0415
**PSMC1**	1.37	0.673	0.0415
**KIAA1671**	-2	0.98	0.0415
**GAL3ST4**	-3.79	1.86	0.0416
**DYNC1I2**	-1.5	0.737	0.0418
**HMOX1**	1.86	0.914	0.0419
**NXF1**	1.19	0.583	0.042
**BRD7**	-1.58	0.776	0.042
**HSD17B4**	-2.37	1.17	0.0421
**PLCXD1**	2.74	1.35	0.0421
**NDUFB2**	1.55	0.764	0.0422
**PHF14**	-1.54	0.759	0.0423
**NECTIN1**	2.91	1.43	0.0423
**RASSF5**	2.97	1.46	0.0425
**PCBP4**	-2.41	1.19	0.0425
**FILIP1**	-1.36	0.669	0.0425
**AC135012.1**	-2.31	1.14	0.0426
**IER5**	1.57	0.774	0.043
**HVCN1**	-3.41	1.69	0.0433
**EIF5A**	1.45	0.717	0.0434
**PBRM1**	-1.75	0.867	0.0436
**IPO7**	1.23	0.609	0.0436
**PLCG1**	-2.62	1.3	0.0436
**ARPC1B**	-1.73	0.861	0.0441
**LHFPL2**	1.85	0.919	0.0442
**IGFBP6**	-1.09	0.544	0.0442
**ARHGAP6**	-2.38	1.18	0.0443
**ITGBL1**	-1.23	0.612	0.0446
**TANGO2**	-3.25	1.62	0.0446
**MRPS24**	1.62	0.805	0.0448
**SLC27A1**	-3.06	1.53	0.045
**GJA1**	1.33	0.666	0.0454
**DSTN**	-0.591	0.296	0.0455
**AATF**	3.23	1.62	0.0457
**BCS1L**	-3.03	1.52	0.0459
**OGFRL1**	2.65	1.33	0.0461

**AK6**	2.35	1.18	0.0463
**LEPROT**	-1.48	0.745	0.0465
**BTN3A2**	-2.46	1.24	0.0472
**TFPI2**	3.37	1.7	0.0474
**XBP1**	1.8	0.907	0.0475
**TPT1-AS1**	-1.92	0.971	0.0476
**ZNFX1**	1.7	0.856	0.0476
**IL1R1**	1.5	0.755	0.0477
**MAP2K7**	-2.26	1.14	0.0477
**PHF21A**	-2.33	1.18	0.0479
**FSTL3**	1.3	0.656	0.0479
**DEGS1**	1.34	0.68	0.0484
**METTL8**	-2.67	1.35	0.0488
**UBA3**	-1.91	0.967	0.0488
**TLE3**	1.89	0.96	0.0489
**AD000090.1**	-1.77	0.899	0.0489
**DNTTIP1**	-2.48	1.26	0.0489
**RAD21**	-2.03	1.03	0.049
**PEAK1**	1.56	0.795	0.0492
**CAVIN2**	-1.93	0.981	0.0493
**EML3**	-1.63	0.83	0.0493
**NR4A2**	1.73	0.88	0.0494
**C11orf91**	3.92	2	0.0495
**TMEM9**	-2.14	1.09	0.0497
**SART1**	3.77	1.92	0.0497
**CALCOCO1**	-1.57	0.8	0.0497
**NFIX**	-0.962	0.49	0.0497
**UGCG**	1.68	0.859	0.05
**CRIP2**	-0.979	0.499	0.05
